# Comparative evaluation of RNA isothermal amplification-gold probe lateral flow assay and targeted next-generation sequencing for the detection of *Mycoplasma pneumoniae* and influenza A and B viruses in children

**DOI:** 10.3389/fped.2026.1741420

**Published:** 2026-03-05

**Authors:** Bin Wang, YangJi Wei, Xiangmei Dong, YanFang Lu, Shiqu Deng, CanWei Chen

**Affiliations:** 1 Clinical Laboratory, College of Clinical Medicine for Obstetrics & Gynecology and Pediatrics, Fujian Children’s Hospital (Fujian Branch of Shanghai Children’s Medical Center), Fujian Medical University, Fuzhou, China; 2 Fuzhou KingMed for Clinical Laboratory Co., Ltd, Fuzhou, Fujian, China; 3 Clinical Laboratory, Fujian Maternity and Child Health Hospital, Fuzhou, China; 4 Clinical Laboratory, Nan'an Hospital, Quanzhou, Fujian, China

**Keywords:** acute respiratory infections, children, influenza virus, *Mycoplasma pneumoniae*, RNA isothermal amplification-gold probe lateral flow technology, targeted next-generation sequencing

## Abstract

**Objectives:**

This study aimed to compare the positive detection rates of RNA Isothermal Amplification-Gold Probe Lateral Flow Technology (RGT) and Targeted Next-Generation Sequencing (tNGS) for *Mycoplasma pneumoniae* (MP) and Influenza A/B viruses in children with acute respiratory infections (ARIs), and to explore their respective advantages and disadvantages.

**Methods:**

Clinical and laboratory data of pediatric patients with ARIs undergoing concurrent RGT and tNGS testing (Jan–Sep 2024) were collected. McNemar's test compared detection rates, and Cohen's kappa coefficient assessed the agreement of the result of between the two methods.

**Results:**

For detecting MP and Influenza B virus, tNGS showed a significantly higher positivity rate than RGT. However, there is no difference between tNGS and RGT in the positive detection rate of Influenza A. The agreement between tNGS and RGT was good for MP detection, but only moderate for Influenza A/B virus detection.

**Conclusions:**

tNGS offers high-throughput, high-sensitivity screening, while RGT is rapid, user-friendly, cost-effective and superior for identifying active infections and monitoring treatment responses. Optimal method selection is dependent on clinical scenarios and diagnostic priorities.

## 1 Introduction

Respiratory infections constitute a significant and pervasive health issue worldwide, closely associated with elevated morbidity and mortality rates (1). According to estimates published in The Lancet (2), deaths from respiratory infections are projected to increase between 2016 and 2040, and the mortality rate is highest among children under 5 years of age and the elderly. Additionally, children under 5 years of age and those of school age 5 years of age and those of school age exhibiting the highest rates of viral positivity and bacterial positivity (3). In “Zhu Futang's Practice of Pediatrics” (9th Edition) (4), Acute respiratory infections (ARIs) in pediatric patients are classified into acute upper respiratory tract infections (AURI), which encompass acute inflammation of the nasal cavity, pharynx, or larynx, and acute lower respiratory tract infections (ALRI) such as acute bronchitis and pneumonia. ARIs have emerged as the predominant cause of hospitalization and mortality among pediatric populations in middle-income countries (5). ARIs are caused by various pathogens, with *Mycoplasma pneumoniae* (MP) and influenza viruses being the most prevalent.

MP is one of the most common causes of respiratory tract infections in children and is the main pathogen responsible for community-acquired pneumonia in approximately 30% of cases involving children (6, 7), it is a pathogenic microorganism without a cell wall, which often causes upper and lower respiratory tract infections. Severe cases may lead to multiorgan dysfunction of the cardiovascular, nervous, and digestive systems (8). The influenza virus, which belongs to the Orthomyxoviridae family, causes influenza and can be divided into three types (influenza A, B, and C). Influenza is a highly infectious acute respiratory disease, and the population is generally susceptible. Previously, the incubation period was reported to be approximately 1–4 days, and the viral positivity rate reached about approximately 50% in children under 5 years of age and in school-age children (9). Additionally, influenza detection varies greatly in different regions and seasons (10) and key differences in diagnostic techniques have rendered comparisons between research findings challenging.

Accurate pathogen diagnosis is crucial for the treatment of ARIs. ARIs are usually diagnosed on the basis of clinical features such as headache, fever, and muscle pain. Routine examinations may uncover non-specific hematological abnormalities and alterations in pulmonary imaging, such as leukopenia, infiltration, or atelectasis. The value of rapid and precise diagnostic methods for ARIs is increasing in clinical practice. Isolation and culture, molecular assays, and serological antibody detection are the primary laboratory diagnostic methods for identifying respiratory pathogens (11). As they have distinct advantages. However, these traditional detection methods are limited in that they can be time-consuming or offer limited sensitivity.

Clinical practice is increasingly emphasizing the importance of rapid and precise diagnostic methods for ARIs. Traditional detection methods, such as microbial culture, are time-consuming, and only small number of the microbial population can be cultured (12). Antigen detection often lacks sufficient sensitivity. Serum antibody detection is incapable of achieving early diagnosis and it is difficult to distinguish between past and acute infections (11). Conventional molecular detection, while highly sensitive and specific, is insufficiently rapid (13, 14). To meet the requirements of clinical practice, rapid diagnostic techniques based on DNA or RNA genomes, such as targeted next-generation sequencing (tNGS) and RNA Isothermal Amplification-Gold Probe Lateral Flow Technology (RGT), have been developed and have subsequently gained recognition for their crucial roles in helping to manage ARIs.

tNGS is a microbe-identification technique based on nucleic acid detection that recently emerged and has since been widely used because of its advantages such as unpredictability and high throughput (15, 16). The main advantage of tNGS is that it can be exceptionally more sensitive than traditional diagnostic techniques, enabling pathogen detection at trace levels.

RGT is an advanced technique that leverages RNA reverse transcription and transcription to achieve RNA amplification, in which, gold-labeled probes are used to detect the resultant RNA amplification products. Because both techniques are based on nucleic acid testing, they offer the dual advantages of precision and rapidity and are gradually gaining traction in clinical practice. However, in clinical practice, their capabilities in detecting respiratory pathogens, as well as their respective advantages and drawbacks, remain poorly understood. Clarifying their differences in pathogen-detection capability will help physicians select the most suitable testing method for their specific diagnostic needs.

In this study, tNGS and RGT were used to detect MP and influenza viruses in patients with ARIs. Our objective was to comprehensively explore their respective advantages and disadvantages of methods for both types of pathogens, specifically in pediatric patients with ARIs.

## 2 Methods

### 2.1 Specimen information

Clinical data and laboratory results were obtained from pediatric patients who presented with clinical diagnosis of ARIs at Fujian Provincial Children's Hospital, who underwent concurrent testing for MP, Influenza A virus, or Influenza B virus using both RGT and tNGS between January 2024 and September 2024. The inclusion criteria were that the patients (1) had an age of <16 years, (2) were diagnosed with an ARI, and (3) underwent concurrent pathogen detection using both techniques with comprehensive clinical documentation available.

### 2.2 Specimen collection

For tNGS, specimens were collected according to the manufacturer's instructions; For MP detection, 95.82% of the specimens were oropharyngeal swabs, and 4.18% were bronchoalveolar lavage fluid (BALF). For Influenza A/B viruses, 95.41% were nasopharyngeal swabs and 4.59% were BALF.

For RGT specimens were collected strictly in accordance with the manufacturer's protocol. Oropharyngeal swabs (100%) were used for MP detection, while nasopharyngeal swabs (100%) were preferred for Influenza A/B virus detection due to higher viral loads in the nasopharynx.

Validation experiments performed in our laboratory confirmed that the DNA/RNA of MP and influenza viruses remained stable for 24 h in BALF, oropharyngeal swabs and nasopharyngeal swabs stored at 2–4°C. Therefore, all clinical specimens in this study were preserved at 2–4°C and subjected to detection within 24 h of collection.

### 2.3 tNGS

tNGS was performed in strict compliance with the manufacturer's instructions (Guangzhou Jinqi Rui Biotechnology Co., Ltd.). Briefly, multiplex polymerase chain reaction (PCR) analysis combined with NGS technology was used to target the highly conserved regions of 198 respiratory pathogens. Specific primers were designed and PCR amplification was performed to enrich for the target pathogens. In a subsequent round of PCR, sequencing adapters were added to enable identification of the source origin. High-throughput sequencing was then performed using a KM MiniSeqDx-CN gene sequencer. The resulting sequencing data were filtered and compared with reference genome sequences to interpret the pathogen-detection results. Sample quality was monitored by simultaneously detecting human DNA as an internal control.

### 2.4 RGT

RGT was performed in strict compliance with the manufacturer's instructions (Wuhan Zhongzhi Biological Technology Co. Ltd.). Briefly, collected samples were lysed using a cell lysis buffer to release pathogen nucleic acids. Subsequently, under the catalysis of reverse transcriptase and T7 RNA polymerase, reverse transcription and transcription occur, enabling the amplification of pathogen nucleic acid fragments. The amplified RNA products are recognized and captured by specific probes in the detection probe solution, leading to the formation of an RNA amplification product-detection probe-gold probe complex, which was fixed onto a nitrocellulose membrane to form visible bands through lateral flow chromatography, enabling pathogen nucleic acid detection. The kit is equipped with an internal reference control for monitoring the processes of sample collection, storage, transportation, and nucleic acid extraction to avoid false- negative results. We used a OneS-16 amplification instrument for these experiments.

### 2.5 Quality control (QC)

RGT assay: the positive QC reaction should yield three distinct bands (T, N, and C bands) on the detection card, whereas the negative NC reaction should produce two bands (N and C bands) only. Notably, paired positive and negative control experiments were performed alongside each sample test to ensure the validity of the results; the QC line (C) and internal control line (N) on the test strip are required to manifest as visible bands. The QC line (C) denotes the validity of the test strip and the procedural accuracy of the entire testing workflow; conversely, the internal control line (N) is indicative of effective nucleic acid amplification, and validates the appropriateness of both the amplification protocol and sample processing steps.

tNGS: In each NGS experiment, internal controls, positive and negative control materials were included. Data quality was required meet the following cutoffs: Q30 ≥ 75%, minimum raw reads ≥ 50 k, normalized internal control reads ≥ 200, or normalized target-region pathogen reads ≥ 3,000.

### 2.6 Data analysis

Statistical analyses were performed using SPSS software (version 24.0; IBM Corp.). Categorical data were expressed as percentages. 95% confidence intervals(CI) for proportions were estimated using the Wilson score method. Differences in positivity rates between the two detection methods evaluated were compared using McNemar's test (paired *χ*^2^ test). Agreement between both methods was assessed using Cohen's kappa. Statistical significance was defined as a two-tailed *P* value of <0.05.

## 3 Results

### 3.1 Clinical information

In this study, we collected data for 2,307 patients, among whom 1,173 underwent concurrent testing for MP using both targeted tNGS and RGT. Within that subset, 48.08% were clinically diagnosed with pneumonia (including severe pneumonia, MP-specific pneumonia, and pneumonia in recovery phase), 14.49% with fever, 11.68% with ARIs, and 24.13% with bronchitis (including acute and related subtypes). The cohort comprised 654 male patients (55.75%) and 519 female patients (44.25%), comprising 475 patients (40.49%) aged 0–3, 399 (34.01%) aged 3–6, and 299 (25.49%) aged ≥ 6 (Table 1). A total of 567 patients were concurrently tested for Influenza A and B viruses via both targeted tNGS and RGT. Their clinical diagnoses included fever (23.10%), pneumonia (39.15%; encompassing severe pneumonia and convalescent-phase pneumonia), ARIs (14.99%), and bronchitis (21.69%; including acute and related subtypes). The population comprised 316 males (55.73%) and 251 females (44.27%). The age distribution was as follows: 239 patients (42.15%) were 0–3, 196 (34.57%) were 3–6, and 132 (23.28%) were ≥6 (Table 2).

**Table 1:** The general characteristics of the MP patients.

MP	Number (*n*)	Constituent ratio (%)
Gender		
Male	654	55.75
Female	519	44.25
Age (years old)		
0–3	475	40.49
3–6	399	34.01
≥6	299	25.49
Clinical diagnosis		
Pneumonia	564	48.08
Bronchitis	283	24.13
ALRI	137	11.68

**Table 2:** The general characteristics of the influenza patients.

Influenza A/B virus	Number (*n*)	Constituent ratio (%)
Gender		
Male	316	55.70
Female	251	44.27
Age (years old)		
0–3	239	42.15
3–6	196	34.57
≥6	132	23.28
Clinical diagnosis		
Pneumonia	222	39.15
Bronchitis	123	21.69
ALRI	85	14.99

### 3.2 Comparing MP-detection rates observed with tNGS and RGT

We tested 1,173 specimens concurrently for MP using tNGS and RGT. Among the specimens tested, tNGS detected 487 cases positive for MP[41.52%, (95% CI: 38.70%–44.34%)], and 686 negative cases[58.48% (95% CI: 55.66%–61.28%)]; BALF samples accounted for 4.18% of the cohort and exhibited a 32.65% (95% CI: 19.66%–45.64%) positive rate for MP, whereas oropharyngeal swabs specimens constituted 95.82% of the total and demonstrated a 41.90% (95% CI: 39.02%–44.78%) positivity rate. No statistically significant difference in positive rates was observed between BALF samples and oropharyngeal swabs (*P* > 0.5).

RGT detected 433 positive cases [36.91% (95% CI: 34.15%–39.67%)] and 740 MP-negative cases [63.09% (95% CI: 60.33%–65.85%)]. When the results were compared with tNGS for concordance, dual-positivity was observed in 408 cases [34.78% (95% CI: 32.06%–37.50%)] for MP and dual-negativity was observed in 661 cases [56.35% (95% CI: 53.51%–59.19%)]. McNemar's test revealed a statistically significant difference in MP-positivity rates between tNGS and RGT analysis (*χ*^2^ = 27.01, *P* < 0.001). Cohen's kappa coefficient analysis demonstrated substantial agreement between both methods [K = 0.814 (95% CI: 0.782–0.846), *P* < 0.001] (Table 3).

**Table 3:** Detection results of MP using tNGS vs. RGT.

Test method	tNGS positive (*n*)	tNGS negative (*n*)	Total (*n*)
RGT Positive (*n*)	408	25	433
RGT Negative (*n*)	79	661	740
Total (*n*)	487	686	1,173

McNemar's test: *χ*^2^ = 27.01, *P* < 0.001; Cohen's kappa coefficient: K = 0.814 (95% CI: 0.782–0.846), *P* < 0.001.

### 3.3 Comparison of influenza A-detection rates between tNGS and RGT

We tested 567 specimens concurrently for Influenza A virus using tNGS and RGT. Among them, 26 cases were positive [4.59% (95% CI: 2.87%–6.31%)] and 541 cases were negative [95.41% (95% CI: 93.69%–97.13%)] for Influenza A virus via tNGS. In contrast, 37 cases were positive [6.53% (95% CI: 4.49%–8.57%)] and 530 cases were negative [93.47% (95% CI: 91.43%–95.51%)] for Influenza A virus via RGT. In addition, 16 cases were consistently positive [2.82% (95% CI: 1.46%–4.18%)] and 520 cases were consistently negative [91.71% (95% CI: 89.44%–93.98%)] after testing with both tNGS and RGT. There was no statistical difference between tNGS and RGT as determined by McNemar's test (*χ*^2^ = 3.23, P > 0.05). Cohen's kappa coefficient analysis demonstrated moderate agreement between the tNGS and RGT methods [K = 0.480 (95% CI: 0.351–0.609), *P* < 0.001; Table 4].

**Table 4:** Detection results of influenza A using tNGS vs. RGT.

Test method	tNGS positive (*n*)	tNGS negative (*n*)	Total (*n*)
RGT Positive (*n*)	16	21	37
RGT Negative (n)	10	520	530
Total (*n*)	26	541	567

McNemar's test: *χ*^2^ = 3.23, *P* = 0.072; Cohen's kappa coefficient: K = 0.480 (95% CI: 0.351–0.609), *P* < 0.001.

### 3.4 Comparison influenza B-detection rates between tNGS and RGT

We tested 567 specimens concurrently for Influenza B virus via tNGS and RGT. Among them, 35 cases [6.17% (95% CI: 4.49%–8.57%)] were positive and 532 cases [93.83% (95% CI: 91.43%–95.51%)] were negative for Influenza B virus via tNGS. In contrast, 20 cases were positive [3.52% (95% CI: 2.01%–5.03%)] and 547 cases [96.48% (95% CI: 94.97%–97.99%)] were negative for Influenza B virus via RGT. In addition, 16 cases [2.82% (95% CI: 1.46%–4.18%)] were consistently positive, and 528 cases [93.12% (95% CI: 91.04%–95.20%)] were consistently negative after testing with both tNGS and RGT. tNGS showed a significantly higher positive rate than RGT, as determined by McNemar's test (*χ*^2^ = 8.52, *P* < 0.05). Cohen's kappa coefficient analysis demonstrated moderate agreement between both methods [K = 0.562 (95% CI: 0.438–0.686), *P* < 0.001; Table 5].

**Table 5:** Detection results of influenza B using tNGS vs. RGT.

Test method	tNGS positive (*n*)	tNGS negative (*n*)	Total (*n*)
RGT Positive (*n*)	16	4	20
RGT Negative (*n*)	19	528	547
Total (*n*)	35	532	567

McNemar's test: *χ*^2^ = 8.52, *P* = 0.004; Cohen's kappa coefficient: K = 0.562 (95% CI: 0.438–0.686), *P* < 0.001.

## 4 Discussion

The present study the first to evaluate RGT, a novel rapid pathogen-detection method, for detecting MP and Influenza A/B viruses in children, by comparing its positive detection rates with those of tNGS. The aim of this analysis was to enhance our understanding of RGT/ tNGS and support its clinical application. Our results indicated that tNGS exhibited higher positivity rates than RGT for MP and Influenza B viruses, whereas there was no difference between tNGS and RGT in the positive detection rate of Influenza A. Both methods showed substantial to moderate agreement. Beyond the comparative positivity rates, the differential detection capabilities of these technologies offer critical insights for their clinical application.

### 4.1 tNGS vs. RGT: molecular traits and clinical implications

tNGS enables high-throughput pathogen identification through either reverse-transcribing RNA for sequencing or directly sequencing DNA (17), while RGT targets the mRNA produced during the reproduction process of the target pathogen. The RNA expression level is associated with infection activity (18), and as a single-stranded molecule, RNA is labile—it exists exclusively in viable pathogens and degrades rapidly following cell death, In contrast, DNA is more stable and can persist in patients for extended periods (19, 20). This fundamental difference determines their divergent clinical implications in assessing infection status. Consequently, our study show that tNGS demonstrated a higher positivity rate for MP and Influenza B viruses detection than RGT, a finding attributable to the ability of tNGS's ability to detect stable, persistently existing DNA even after pathogen inactivation—thus its positive results may include cases of past infection or non-viable pathogen carriage—whereas RGT only recognizes labile RNA from viable pathogens, with its positive results specifically indicating active pathogen replication and current active infection. Thus, the lower positivity rate of RGT compared with tNGS should not be interpreted as inferior analytical performance, but rather as a reflection of its biological specificity toward active infection, a feature that confers great clinical value and is particularly advantageous in pediatric patients (in whom avoiding unnecessary antimicrobial exposure is paramount). Furthermore, RGT can guide timely anti-infective treatment: a conversion from RGT-positive to negative during treatment suggests effective therapy due to inhibited or eliminated pathogen replicative activity, enabling clinicians to adjust treatment regimens.

Beyond this, the ability of tNGS to detect persistent pathogen DNA enables infection detection even in late disease stages with degraded RNA (21). A positive tNGS result may indicate active infection, past infection (residual DNA), or asymptomatic colonization, carrying dual-edged clinical significance. Its high sensitivity is invaluable for refractory cases (e.g., severe pneumonia of unknown etiology), detecting trace nucleic acids missed by other methods—for example, identifying MP DNA wherein RGT is negative but symptoms persist, prompting consideration of early/late-stage infection or mixed pathogens. However, tNGS cannot distinguish active from inactive pathogens, raising the risk of overdiagnosis and overtreatment (22). Importantly, the clinical relevance of a diagnostic assay should not be evaluated solely based on its positive detection rate. In the context of respiratory infections, clinicians are often more concerned with whether a detected pathogen represents an active, ongoing infection that requires therapeutic intervention, rather than the mere presence of residual nucleic acid.

Previous reports have demonstrated that MP nucleic acid loads were significantly higher in BALF specimens than in oropharyngeal swabs samples (23–25). Suggesting BALF should yield higher MP positivity rates. In the present study, tNGS detected lower MP positivity in BALF than in oropharyngeal swabs, but this difference was not statistically significant. Several factors may account for this discrepancy. First, the limited number of BALF specimens utilized in the present stud may not adequately represent the true diagnostic yield of BALF and may introduce sampling bias. Second, variability in clinical sampling procedures—including the timing of bronchoscopy relative to disease course, lavage volume, and specimen handling—may affect nucleic acid recovery and detection sensitivity. Third, BALF specimens the present study were primarily submitted for tNGS, whereas oropharyngeal swabs were uniformly used for RGT, which may introduce pre-analytical heterogeneity when comparing detection rates across specimen types and methodologies. Future studies should increase the BALF sample size, implement stringent pre-analytical and intra-analytical quality control, and conduct external validation to corroborate these findings.

### 4.2 Analysis of influenza A/B virus detection

For influenza virus, tNGS exhibited a significantly higher positive rate for Influenza B than RGT, though the absolute discrepancy was clinically negligible with moderate inter-method agreement. Despite the theoretical sensitivity advantages of the tNGS (26, 27). No difference in Influenza A positivity rates was observed. The main reason lies in the present study's non-epidemic timing leading to extremely low influenza positivity. This epidemiological context likely reduced the statistical power to detect differences between RGT and tNGS, particularly for Influenza A virus, for which no significant difference in detection rates was observed. However, according to the findings of the present study, RGT thus emerges as a cost-effective, rapid option for routine influenza screening in non-epidemic periods, while tNGS remains valuable for high-sensitivity scenarios (e.g., epidemiological surveillance), this is also one of the findings of the present study. In future studies, influenza seasonality should be considered and the positive sample size should be increased to more accurately assess the comparative diagnostic performance of RNA- and DNA-based detection methods for influenza viruses.

### 4.3 Comparative advantages, limitations, and clinical scenarios

In terms of methodologic characteristics, tNGS requires specialized personnel, stringent laboratory infrastructure, and several hours of turnaround time (28). Its potential low specificity may detect normal biome or transient colonizing pathogens (not causative of ARIs), and interpreting low-abundance pathogens is challenging due to colonization/contamination signals—demanding greater proficiency from clinicians to ensure accurate diagnosis and treatment planning (26). Unlike conventional tNGS and RT-PCR, RGT offers a user-friendly workflow with minimal technical barriers, relaxed environmental requirements, and a turnaround time of tens of minutes—suitable for routine laboratory use. It utilizes pathogen-specific primers to amplify unique RNA sequences, ensuring high diagnostic specificity, and presents results in a clear, accessible format for clinicians and patients. RGT is more cost-effective than tNGS and is increasingly adopted in primary hospitals and grassroots medical institutions.

Therefore, while tNGS excels in broad and highly sensitive pathogen screening, RGT offers unique clinical value by preferentially identifying transcriptionally active pathogens, thereby providing rapid, actionable information for clinical decision-making.

### 4.4 Study limitations and future directions

Several limitations of the present study should be acknowledged. First, the lack of a gold standard diagnostic method (there is no universally recognized gold standard for molecular biological detection) precludes the calculation of definitive sensitivity and specificity for either RGT or tNGS. Our comparison is thus relative. Second, as noted, the study period coincided with a non-epidemic phase for influenza, resulting in low positivity rates that may limit the statistical power and generalizability of the findings for viral detection. Third, the low proportion of BALF specimens limits the robustness of conclusions regarding specimen type comparisons. We have started conducting multi-center studies conducted across different epidemiological seasons and incorporating a higher yield of lower respiratory tract samples is warranted to validate and extend our findings.

## 5 Conclusion

In terms of the positive detection rate, tNGS demonstrated superior detection for MP and Influenza B viruses compared to RGT. However, the selection of these two methods should not be based on sensitivity alone but individualized according to clinical context, the suspected pathogen, and the clinical question to be addressed (e.g., comprehensive screening vs. confirmation of active disease). Specifically, tNGS offers the advantages of high-throughput and highly sensitive comprehensive pathogen screening, while RGT enables rapid, cost-effective, and user-friendly detection of specific pathogens, and is particularly valuable for identifying active infection, guiding timely antimicrobial therapy, and monitoring treatment responses. In conclusion, although tNGS had higher detection rates for certain pathogens, RGT confers distinct clinical benefits by reflecting active infection status via RNA detection. Collectively, these two assays should be regarded as complementary tools in clinical practice, where RGT addresses the clinically critical question of confirming active infection, while tNGS excels in initial comprehensive pathogen screening.

## Data Availability

The raw data supporting the conclusions of this article will be made available by the authors, without undue reservation.
